# Complete duplication of the penis - A case report

**DOI:** 10.1016/j.eucr.2021.101892

**Published:** 2021-10-14

**Authors:** Saidanvar Agzamkhodjayev, Kobiljon Ergashev, Zafar Abdullayev, Asqar Soliyev, Ruslan Batrutdinov

**Affiliations:** a Department of Urology, National Children's Medical Center, Tashkent, Uzbekistan; b Department of Urology, Tashkent Pediatric Medical Institute, Tashkent, Uzbekistan; c Scandinavia Clinic, St Petersburg, Russian Federation

**Keywords:** Penile duplication, Diphallia, Congenital anomaly, Ultrasonography of the kidneys ureters and bladder, (USG KUB)

## Abstract

Penile duplication is a very rare urological entity. It may be associated with other congenital conditions such as urogenital, GI tract and musculoskeletal anomalies. Properly classifying the condition may dictate the final treatment options. Our current case is the complete true duplication in which we performed side-to-side urethra-urethral anastomosis. We spared the posterior urethra as it may end up with postoperative urinary incontinence.

## 1 Introduction

Duplication of the penis is a rare anomaly with the incidence of 1 in 5 or 6 million. It may be presented as isolated or in part with other congenital urogenital anomalies as ectopic or horseshoe kidney and bladder duplication, GI tract abnormalities as imperforate anus, and also problems with musculoskeletal field as pubic diastasis and lumbosacral anomalies.^1^ The index case was also associated with anal atresia which was operated previously right after birth. Although each penis is structurally normal, it is a great surgical challenge for the surgeon. In this paper we report a case of complete penile duplication and its surgical management in a 7-year-old boy.

## 2 Case report

A 7-year-old male child was referred to our clinic with abnormal genitalia. Initial physical examination identified two completely developed penises with common shaft skin (Fig. 1A). He was fully continent and there was a urine flow from both urethras. On his first day of life, proctoplasty was performed to fix the anal atresia. MRI (Fig. 1B) showed two completely developed penises with each having separate two cavernous bodies, one spongious body. Abdominal USG and CT urography showed normal KUB on each side with an aberrant vessel to the right kidney. Voiding cystourethrogram (VCUG) showed two separate urethras opening into the single bladder via a single bladder neck without any signs of reflux. Uroflowmetry studies were performed through right and left penises separately with normal Q_max_ - 11.8 and 10.3 ml/s, respectively.

**Fig. 1A:**
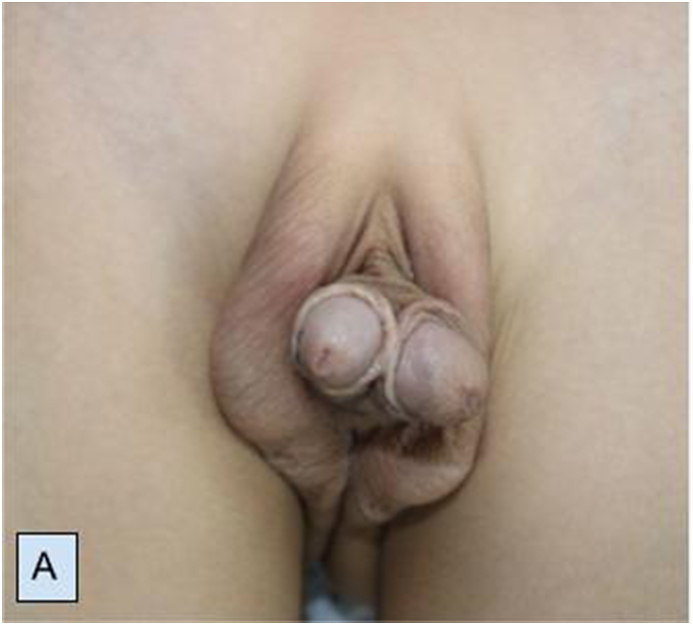
Two completely normally developed penises with a common shaft.

**Fig. 1B:**
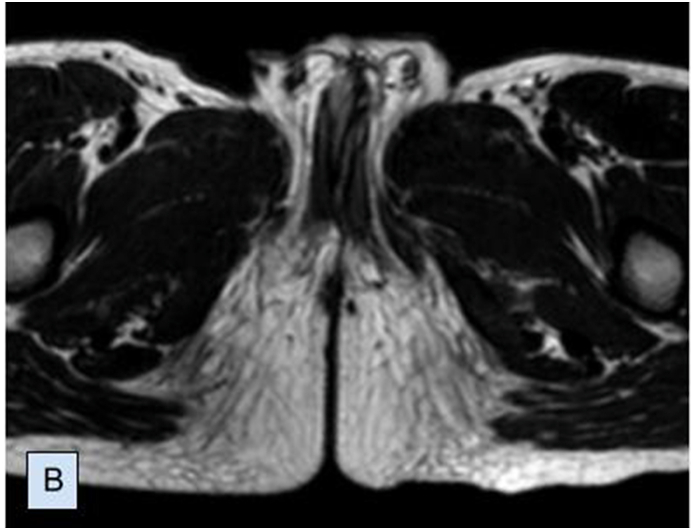
MRI study of the pelvic organs including penile structures.

Preoperatively, retrograde urethrogram (Fig. 1C) and cystourethroscopy were performed through both urethrae and VCUG findings were confirmed. Interestingly, verumontanum was common for both urethras (Video 1). We performed left partial penectomy (Fig. 2A), side-to-side urethra-urethral anastomosis in anterior urethra. The latter was achieved between spatulation of the proximal end of the left penile urethra and the side of the proximal part of the right penile urethra. Two catheters were placed: one into the bladder to drain the urine postoperatively (percutaneous cystostomy) (Fig. 2B); second through the right urethra and across the anastomosis. Urethral catheter was removed on the 10th and cystostomy catheter was removed on the 21st postoperative days.

**Fig. 1C:**
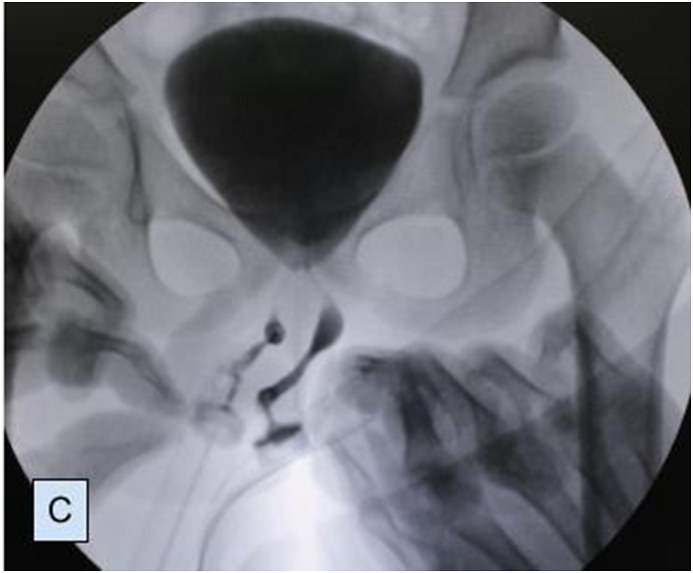
Intraoperative retrograde urethrogram.

**Fig. 2A:**
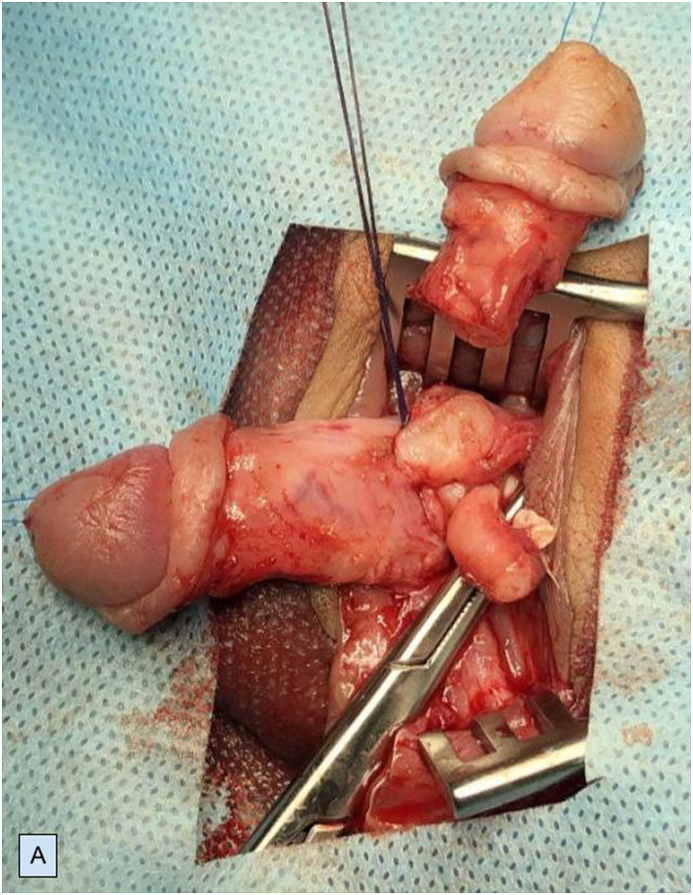
Partial left penectomy.

**Fig. 2B:**
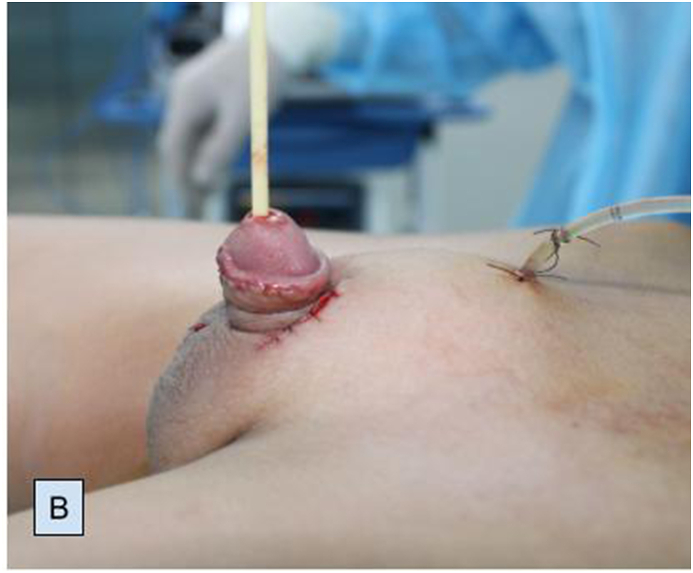
Two catheters: urethral and cystostomy tubes.

Supplementary video related to this article can be found at https://doi.org/10.1016/j.eucr.2021.101892

The following is the supplementary data related to this article:

Video 1 Cystoscopic view of both urethras and common verumontanum.1 Video 1

The patient was fully continent with normal voiding and postoperative two months uroflow study was performed revealing normal Q_max_ of 14.2 ml/s (Fig. 2C). Informed consent was obtained from the parents of the patient.

**Fig. 2C:**
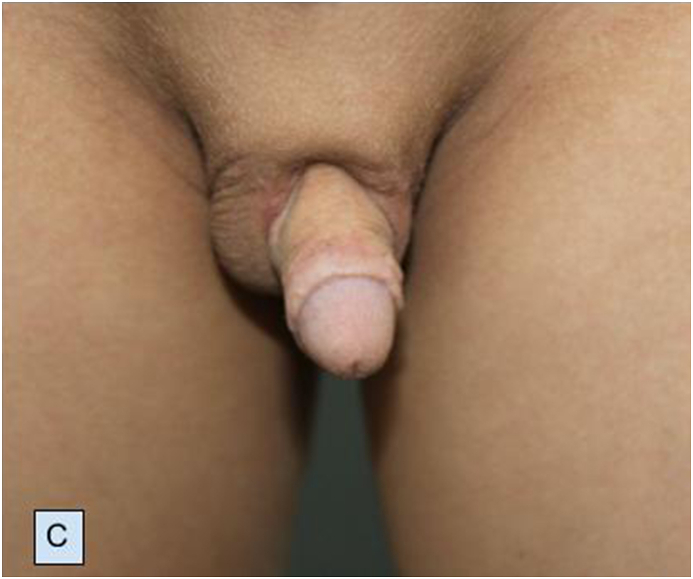
Postoperative two months appearance.

## 3 Discussion

Penile duplication is a rare congenital anomaly with the incidence of 1 in 5–6 million. Although the anomaly is known from the XVII century, there are only just over 100 cases reported so far.

Most of the penile duplication cases are associated with urogenital, anorectal malformations and musculoskeletal anomalies. Associated urogenital anomalies include bladder exstrophy, bladder duplication, bifid scrotum and renal anomalies.^1^

Key mechanism behind the embryogenesis of penile duplication is indefinite, but can be explained with: (1) “separation” of the pubic tubercles during embryogenesis, in which each phallus has one corporal body and urethra, or (2) “cleavage” of the pubic tubercle in which each phallus has 2 corporal cavernous bodies and urethras.^2^

Gyftopoulos et al.^3^ proposed a classification where cases can be divided into 2 broad categories: true diphallia and bifid phallus. Both can be subclassified into partial or complete duplication. True complete diphallia will have 2 well-developed penises (with 2 corpora cavernosa and 1 corpora spongiosum).^4^ True partial diphallia will have a smaller or rudimentary duplicate penis (with complete structures that is 2 corpora cavernosa and 1 corpora spongiosum). If the duplicate penis does not have all the structures, for example one corpora cavernosum they are classified as bifid phallus. Depending on the degree of separation, bifid phallus is further subclassified into complete and partial. Complete bifid phallus has separation at the base whereas, partial bifid phallus has separation at the glans.

According to Gyftopoulos’ classification, our case has true complete diphallia.

We performed side-to-side anastomosis between anterior parts of urethras, in the proximal penile part. This resulted in structural and functional urethra. In order to maintain the continence postoperatively, our dissection did not include the posterior urethra.

Diphallia is a rare congenital anomaly that can be associated with urogenital, anorectal malformations. Hence the presentation of the patients may be different and requires an individualized approach. Side-to-side urethral anastomosis with avoiding dissection in the posterior urethra results in a satisfactory outcome.

## Authors contribution

KE: Conceptualization, Investigation, Resources, Writing - Original Draft.

ZA: Conceptualization, Methodology, Data Curation, Supervision.

SA: Supervision. All authors have read and approved the final manuscript.

## Declaration of competing interest

The authors declare no conflicts of interest.

## References

[1] Karagözlü A.A., Uçar M., Çelik F., Kırıştıoğlu İ., Kılıç N. (2018 Jul 24). Complete penile duplication with structurally normal penises: a case report. Balkan Med J.

[2] Bhat H.S., Sukumar S., Nair T.B. (2006). Successful surgical correction of true diphallia, scrotal duplication, and associated hypospadias. J Pediatr Surg.

[3] Gyftopoulos K., Wolffenbuttel K.P., Nıjman R.J. (2002). Clinical and embryologic aspects of penile duplication and associated anomalies. Urology.

[4] Gupta M., Virdi V.J.S. (2016). Rare case of isolated true complete diphallus – case report and review of literature. SAS J. Surg..

